# Supplementary material for: The possibility of integrating motile sperm organelle morphology examination (MSOME) with intracytoplasmic morphologically-selected sperm injection (IMSI) when treating couples with unexplained infertility
Source: PLoS One. 2020 May 1;15(5):e0232156. doi: 10.1371/journal.pone.0232156 (PMC7194411; doi:10.1371/journal.pone.0232156)
Supplement: Attachment — Submitted filename: Response to reviewers.docx [file pone.0232156.s003.docx]

1. Major: Criticism of many unexplained infertility studies stems from differences in inclusion/exclusion criteria for ‘unexplained’ couples. Additional detail in the materials and methods on page 4 (or supplement) regarding the ovulatory, male factor, uterine/tubal/endometriosis, and ovarian reserve workup of the cases and controls would strengthen the ability of the reader to interpret generalizability to their unexplained infertility/mild male factor population. In the discussion it is recognized as a limitation that this level of detail appears unavailable in the control group. But is the case group all unexplained infertility or would some of them qualify as male factor fertility? The novelty of this study is using the technique in an unexplained infertility population rather than male factor infertility, so an ability to differentiate between them is critical to the claim of novelty.

Thank you for your comment.

All the included couples in the case group and control group had unexplained infertility, and had experienced at least three failed IUI cycles and at least one failed IVF-ICSI cycle. Unexplained infertility was an inclusion criterion. The missing data in the control group was not related to the diagnosis of unexplained infertility or the treatment they underwent.

According to a recent manuscript published in Human Reproduction, subfertility was defined as “couples trying to conceive for at least 12 months. Selected subfertile couples had regular menstrual cycles, at least one patent fallopian tube and a total motile sperm count >1 × 10^6”^ (PMID [30395266](https://meir-tdnetdiscover-com.meir-ez.medlcp.tau.ac.il/logging/outgoing?url=https%3A%2F%2Fwww.ncbi.nlm.nih.gov%2Fpubmed%2F30395266&key=718b9ea8-a589-4e42-a247-148ba8ab0307)), which enabled them to try IUI. Therefore, they can be added to the unexplained infertility group. I added the definition of male subfertility to the methods section, page 4. “Male subfertility was defined as a total motile sperm count >1 × 10^6”^ [8].”

We emphasized this in the methods section and added exclusion criteria, pages 4,5.

“Exclusion criteria were Mullerian abnormality or submucosal or subserosal fibroids >7 cm, despite regular menstrual cycles, because of the negative effect of these complications on implantation and pregnancy outcomes [9]; ovulation abnormalities; tubal pathology; and male infertility (when the total motile sperm count was <1 × 10^6^, because IUI treatment does not benefit this group).”

2. Major: studying the pregnancy rate per couple has some advantages, but additional detail regarding the representation of fresh/frozen embryos/numbers of transfers would be appreciated. Additionally, the length of follow-up per couple is inherently different over an overall 8 year timespan. Consider reporting average length of treatment in couples? Do I assume correctly that only the first pregnancy represented by the embryos generated in an IMSI cycle would be represented in the data? Additionally, how was pregnancy defined in your electronic database? Home test, serum?

The pregnancy rate represented in the data is the cumulative pregnancy rate (pregnancy from fresh and frozen embryos). We included the results of the first IMSI only, because we assumed that if there was a hidden male factor it would be resolved in the first IMSI cycle.

I added the definition to the methods section, page 5: “Clinical pregnancy, defined as missed abortion, blighted ovum or fetal pole with heartbeat, was considered a positive result. Chemical pregnancy was considered a negative result”.

3. Major: I would presume the comparison of outcomes between controls collected 2010-2018 and cases 2015-2018 would generally find that controls had lower pregnancy/delivery rates, at least with US trends; I don’t know if the same can be extrapolated to other countries. What would a sensitivity analysis comparing the controls from 2015-2018 to cases from 2015-2018 show? On a related question, how was the decision made to include the number of cases you did? Was a power analysis done, or was it a convenience sample based on introduction of an EMR in 2010 and including all cycles that met criteria?

I added the following explanation to the statistical analysis section. Page 8.

“Based on the assumption of a 12% difference in pregnancy rates between groups, we calculated that a sample size of 500 couples would be sufficient to provide a power of 80% to show an effect, at two-tailed alpha of 5%.”

We assumed that the pregnancy rate among couples with unexplained infertility who failed a previous IVF-ICSI and had a hidden male factor would be similar to that of couples with their first treatment, which is described in the literature as 40-45%. The pregnancy rate among the control group is 28%, therefore, the difference we used in calculating the sample size was 12%.

The control group included 285 couples, in which only 61 were in 2015-2018, because of the small sample size we included cases from 2010-2015. The cumulative pregnancy rate among those 61 cases was 24.6%, delivery rate 8.2%, abortion rate 16.39%.

4. Major: The title does not inform the reader of the study design, population, or intervention of this study, and the eight-year follow-up was only for the controls, not the cases. Title revision strongly recommended (consider consulting the STROBE guideline).

Thank you for your comment. We changed the title to:

“The possibility of integrating motile sperm organelle morphology examination

(MSOME) and intracytoplasmic morphologically-selected sperm injection (IMSI) in

treating couples with unexplained infertility: a historical, case-control study”

5. Minor: I’m not sure I understand/agree with the logic behind the ‘explanation for this apparent contradiction…that a lower percentage of normal cells in MSOME necessitates an extensive search under ultra-magnification to find the highest quality spermatozoa.’ The criteria by which ‘highest quality’ are judged would not seem to be different in group A and B, but the extensiveness of the search has been a major criticism of the IMSI technique and a concern with the time the oocytes wait to be injected.

The maximum time from providing the semen sample to oocyte injection is four hours. Couples with higher percentage of normal cells at MSOME do not gain benefit from the extensive search, they have enough normal sperm for fertilization.
In addition, men with higher percentage of normal cells in the ejaculate can be managed with routine IVF ICSI . But, when there are only a few normal spermatozoa in the ejaculate, the chance of finding them in routine IVF ICSI is low. I added this explanation to Page 15.

6. Minor: Bonferroni corrections used for multiple comparisons is great, but it’s not clear what alpha was used to judge significance in the different cases; I like to be able to see how close the p-value is to the alpha.

The alpha used for the Bonferroni Correction was 0.05/3=0.017. In pregnancy rates between groups A and C, p=0.002. In delivery rate between groups A and C, p=0.007, and for groups A and B, p=0.023. For total motile counts between groups A and B p=0.005, between groups A and C p=0.011. I added this to the statistical analysis section, Page 8.

7. Minor: is the IMSI technique success related to the embryologist/andrologist examining the sperm? I can’t imagine a single embryologist/andrologist performed all the studies, was there a bias in the most experienced performing those for Group A where difficulty in finding normal sperm was anticipated?

We have 5 embryologists in our lab. Each one examines the sperm randomly. That means that all 5 embryologists examined each group in the study, without any particular preference.
