# Supplementary material for: The possibility of integrating motile sperm organelle morphology examination (MSOME) with intracytoplasmic morphologically-selected sperm injection (IMSI) when treating couples with unexplained infertility
Source: PLoS One. 2020 May 1;15(5):e0232156. doi: 10.1371/journal.pone.0232156 (PMC7194411; doi:10.1371/journal.pone.0232156)
Supplement: Attachment — Submitted filename: Response to reviewer 23-02-2020.docx [file pone.0232156.s004.docx]

Marco Aurélio Gouveia Alves

Academic Editor

PLOS ONE

Re: PONE-D-19-23244R1: The possibility of integrating motile sperm organelle morphology examination (MSOME) with intracytoplasmic morphologically-selected sperm injection (IMSI) when treating couples with unexplained infertility

Dear Professor Alves,

Please find below, our response to the additional comments of reviewer 2. Thank you.

**Reviewer #2**

**1. One of my concerns is why authors make a difference between MSOME and IMSI. MSOME is not a previous procedure that is part of the process of IMSI? When you read the title and the manuscript it seems that there are 2 different procedures.**

Thank you for your comment. MSOME and IMSI are two different procedures. MSOME is an advanced semen analysis that examines the morphology of motile spermatozoa under ultra-magnification of 6300x. It is the process of looking for normal spermatozoa using an inverted light microscope equipped with high-power Nomarski optics, enhanced with digital imaging to achieve magniﬁcation up to 6300×. IMSI is the micromanipulation of the spermatozoa found after extended search and injected into the oocyte.

**2. Is control group a real control? I am concern about this topic. The only information that we have about them is the reproductive outcome, but we don´t know nothing about the % or normal spermatozoa or the rest of seminogram values. Authors should consider delete this group and built the manuscript discussion making the comparison between groups A, B and C. As they stated: group C is the normal/average group and further comparisons should be done with this group. What we know about the proposed control group?**

Thank you for your comment. According to your recommendation we deleted the control group.

**3. Authors show beneficial effect of IMSI versus ICSI. This expected result agrees with previous reports that highlight the beneficial outcome of IMSI. Nevertheless, it is hard to understand why the group with higher percentage of anormal spermatozoa had the higher reproductive outcome. The only reason that could explain this contradiction is that the physician made bigger effort to find a normal cell and performed later the ICSI. Once you finish to read the manuscript the conclusion that the reader made is that IMSI is beneficial for those patients with severe morphological sperm problems. But since physician are selecting normal spermatozoa why the rest of group are not improving? Bigger effort should be made to explain this contradictory result.**

You are correct, this concept is difficult to understand. Our cohort included couples who had at least one unsuccessful, conventional IVF-ICSI cycle, assuming that moderate and mild male factor abnormalities can be solved in the first IVF cycle (enough normal spermatozoa in the ejaculate). Failure to achieve pregnancy in the first or second cycle is not due to unresolved male factor and therefore an extended search will not enhance the implantation rate. In contrast, with severe teratozoospermia requires an extended search to be resolved, failure in the previous cycles can be explained by an unresolved, unknown male factor. In support of this assumption, a previous study found an increase in implantation and pregnancy rates using IMSI over conventional ICSI for patients with previously unsuccessful ICSI, with no difference in the results of the first cycle with ICSI vs. IMSI (15. Klement AH, et al. Intracytoplasmic morphologically selected sperm injection versus intracytoplasmic sperm injection: a step toward a clinical algorithm. Fertil Steril 2013;99:1290-3. doi: 10.1016/j.fertnstert.2012.12.020).

This is explained in page 12, second paragraph.

**4. Minor comments:
Introduction Section:
Please delete this sentence “One study found that ICSI resulted in a similar, cumulative live birth rate as compared with IVF for couples with non-male factor infertility [7]” It doesn´t apport information.**

We deleted the sentence.

**5. Material and Methods:
Please rephrase it: All couples underwent MSOME when IMSI was done within 6 months of the MSOME. What this means you performed a MSOME analysis and later before 6 months you performed an IMSI. Or maybe did you make a MSOME and you freeze the spermatozoa and later or you performed an IMSI. I am sure this is not the message that you want to send but it is rather confusing. Please clarify.**

MSOME is the examination of the semen. According to this examination, we divided the cohort into 3 groups according to the percentage of normal spermatozoa in the semen. In our cohort, we included only couples who had MSOME and proceeded to IMSI within 6 months from the MSOME.

Couples who want to proceed to IMSI provide a new semen sample on the day of oocyte pick-up. This was clarified in the Abstract and Methods.

**6. When you wrote “IVF/ICSI cycles. Male subfertility was defined as a total motile sperm count >1 × 106” What you really mean is less than a million?**

Male subfertility was defined as a total motile sperm count ≥1 × 10^6^.

Male infertility was defined as a total motile sperm count was <1 × 10^6^

**7. Please defined what do you consider “Chemical pregnancy”.**

When the level of hCG is initially elevated enough to produce a positive result on a pregnancy test but then declines before a gestational sac is detected with [ultrasound](https://www.medicalnewstoday.com/articles/245491.php).

I added this to the Methods section, page 5

**8. When you described the sperm selection “The upper liquid was then removed and the pellet was re-suspended in Quinn's Sperm Washing Medium (Sage In-Vitro Fertilization, Inc.) and centrifuged again for 5 minutes at 600 rpm at room temperature. “What do you mean for the pellet? The spermatozoa that can be found on the bottom of the tube? Do you take the spermatozoa and semen contaminants that can be found on the 40% fraction? Please specify this point.**

The pellet is the cellular fraction at the bottom of the tube. Generally, the semen itself will remain above the upper layer and debris and immotile cells will get "stuck" between the upper and lower layers. Most of the motile cells will pass through both layers and reach the bottom of the tube, forming the pellet.

**9. It is possible to insert in table-3 information about the concentration of the ejaculates. It is the only reason that can explain why different groups that have the same % of motile spermatozoa and same ejaculate volume have different values of total motile count.**

The difference in total motile count is that this parameter was calculated after sample preparation in which spermatozoa are separated based on density gradient, where morphologically normal spermatozoa have higher densities. Couples with low percentage of normal cells at MSOME, have high percentage of spermatozoa with severe morphological abnormalities; thus, fewer motile spermatozoa are extracted.

This was explained on page 14.

**10. Statistical analysis:
Please explain the assumption of a 12% difference in pregnancy rates between groups. How did you calculate this? There is a big gap between the number of couples included in each group. Did the author have this in mind when they performed the statistic? Did you balance statistically this difference?**

We did this calculation in order to estimate the sample size of the study group and the control group. Based on your previous comment we deleted the control group.
