# Supplementary material for: The possibility of integrating motile sperm organelle morphology examination (MSOME) with intracytoplasmic morphologically-selected sperm injection (IMSI) when treating couples with unexplained infertility
Source: PLoS One. 2020 May 1;15(5):e0232156. doi: 10.1371/journal.pone.0232156 (PMC7194411; doi:10.1371/journal.pone.0232156)
Supplement: Attachment — Submitted filename: Response to reviewer.docx [file pone.0232156.s005.docx]

**Response to reviewer-**

**Page 2: ‘2010-2018’. Now that the control group is removed, isn’t the study only 2015-2018?**

Thank you for your comment. You are right. I changed this in the abstract.

**Page 3: ‘Using this technique together with a micromanipulation system has allowed the introduction of a modified intracytoplasmic sperm injection (ICSI) procedure, known as intracytoplasmic morphologically-selected sperm injection (IMSI)’. Can you say something about ICSI in the second paragraph to introduce this more familiar acronym sooner? Introducing it in the current state is confusing.**

I added a paragraph in page 3 explaining the difference between ICSI and IMSI: "When the spermatozoa with normal morphology and motility selected for ICSI are detected under a magnification of ×400, in IMSI the motile spermatozoa are selected under magnification up to 6300×[5]".

The advantage of IMSI is that the sperm is detected under magnification up to 6300× which can detect subtle organellar malformations in motile spermatozoa that an embryologist might consider normal for fertilization at 200× to 400× magniﬁcation (ICSI). This explanation is written in page 3 and page 12.

**Page 4: ‘All couples underwent MSOME when IMSI was within 6 months of the initial MSOME’. This is unclear.**

I changed this sentence to: All couples underwent IMSI within 6 months of the initial MSOME.

**Page 4: ‘at least one failed IVF-ICSI cycle’ vs ‘several, previous failed IVF/ICSI cycles’. To whom was MSOME offered, and who was included in the study? Also if IVF/ICSI and IVF-ICSI the same, recommend consistency.**

MSOME was offered to couples who had at least one failed IVF-ICSI cycle. I changed this. And changed IVF/ICSI to IVF-ICSI.

**Page 10: ‘Group A 10 included 55 cases in which no normal cells were found at MSOME, but normal cells were found during IMSI at ovum pick-up for 49 (89.1%). Group B included 184 couples and Group C 32 couples.’ Did all Group B and Group C patients have normal cells found for IMSI? If not, an argument could be made that maybe MSOME is just not predictive of IMSI, even if <6 months, so I would include the information if you have it.**

All the couples in group B and C had normal cells for IMSI.

**Page 12: consider modifying as bolded: ‘Thus, MSOME can detect subtle organellar malformations in motile spermatozoa that an embryologist might consider normal for fertilization at 200× to 400× magnification [5], especially important when a normal sperm nucleus is a significant factor for successful implantation and pregnancy in ICSI procedures [13,14].**

I did this.

**Page 13: the argument is strengthened, but make sure formatting (IVF-ICSI or IVF/ICSI or IVF ICSI) is consistent.**

Thank you. I changed to IVF-ICSI.

**Page 13: It is confusing to have 2 definitions for ‘male subfertility’: both >1 million cells and Group B.**

All the couples in the cohort had unexplained infertility or male subfertility, when male subfertility is defined as a total motile sperm count ≥1 × 10^6^ (written in page 4), that means that each group may have cases of male subfertility.
